# Health-Outcomes and Epidemiologic Research of Transgender Patients Requires Simple, Meaningful Diagnostic Codes: A Retrospective Review of California Emergency Department Visits

**DOI:** 10.26502/jsr.10020351

**Published:** 2024-03-14

**Authors:** Joshua Sterling, Aleksandra Golos, Sandeep Sandhu, Maurice M Garcia

**Affiliations:** 1 Department of Urology, Yale School of Medicine, New Haven, CT, USA; 2 Department of Obstetrics and Gynecology, Cedars-Sinai Medical Center, Los Angeles, CA, USA; 3 Department of Urology, Cedars-Sinai Medical Center, Los Angeles, CA, USA; 4 Cedars-Sinai Transgender Surgery and Health Program, Los Angeles, CA, USA

**Keywords:** ICD codes, Sex and gender minority populations, Electronic health record, Transgender health

## Abstract

**Purpose:**

We assessed the frequency of emergency department (ED) visits by transgender individuals, examined whether these visits were related to gender transition healthcare, and identified longitudinal trends in relevant International Classification of Disease (ICD) codes.

**Methods:**

We conducted a retrospective review of aggregated patient records using the California Office of Statewide Health Planning and Development database. ED visits from 2012–2021 that involved ICD-9(2012 to Q3 2015) or ICD-10(Q4 2015 to 2021) codes commonly associated with transgender patients were identified, examining trends in ICD code usage.

**Results:**

We identified 393 relevant ED visits (0.0037% of all visits) in 2012, compared to 2642 visits (0.021% of all visits) in 2021. This represents a 570% increase in ED visits by transgender individuals, despite only a 22% increase in ED visits overall. Gender identity disorders were the primary reason for seeking care in 0.76% of relevant visits in 2012, compared to 3.0% in 2019. The use of diagnosis codes for “transsexualism” decreased from 93% of visits in 2012 to 52% in 2021; the use of codes for “gender identity disorder” increased from 43% of visits in 2016 to 47% in 2021.

**Conclusions:**

This is the first attempt to assess transgender healthcare needs using a non-LGBT-specific database, providing insights for clinical and policy decision-making. The significant increase in the usage of gender-identity disorder diagnosis codes suggests that the prevalence of gender dysphoria is severely under-estimated. Better documentation practices are needed to improve care and track health and epidemiologic outcomes for transgender patients.

## 1. Introduction

During the last several years, there has been a steady increase in the visibility of the transgender (TG) and gender non-binary (GNB) communities. This coincides with changing social mores and greater social acceptance of gender diversity, as well as greater access to transgender care under state Medicaid, federal Medicare, and commercial insurance programs [1]. Despite this increased public awareness, sex and gender minority populations remain among the most marginalized and underserved populations in medicine [2]. It is well documented that there are numerous barriers to healthcare for TG patients, including patient reluctance to disclose, lack of provider experience and education, and healthcare systems and electronic health records (EHR) that are not welcoming for TG patients and often exacerbate gender dysphoria [3,4].

Estimates of the prevalence of gender dysphoria in the US are inconsistent due to differing methodologies; conservative estimates suggest that 0.3% of the population is TG, while other surveys have found that as many as 2.7% of responders self-identify as TG or GNB [5,6]. The most reliable data estimates that 0.6% to 0.7% of the US population, and 0.76% of the population of California, identifies as TG [7]. A challenge to identifying patients as TG and GNB in the healthcare setting is that many providers often do not query gender or sexual identity [8]. Furthermore, large population-based databases are not adept at capturing TG or GNB individuals for several reasons: variations from state to state in requirements to officially change one’s gender, slow adoption in the US to include preferred names and pronouns, assigned sex at birth and gender identity in the EHR, and a lack of training for the nursing and support staff who register patients [2,9,10].

These barriers are further complicated by the multitude of different International Classification of Disease (ICD) codes for “transgender” and “gender non-binary.” This can be traced to the fact that the ICD codes in use today were designed for use by mental health professionals, and while different codes may reflect important mental health history nuances, providers without a background in mental health (or knowledge of the patient’s mental health history) are forced to select diagnoses among different ICD diagnosis codes, all of which might appear similar to them. With many different codes in use, it is less straightforward to identify which patients are TG (let alone those which are GNB) in patient care databases. To the best of our knowledge, this is the first study to look at diagnosis codes to investigate healthcare usage for this population.

The purpose of this study was to use ICD-9 and ICD-10 diagnosis codes to estimate the frequency that TG people interact with the healthcare system [11,12]. Secondarily, we wanted to assess whether their primary reason for seeking medical attention was related to their transition, and finally, to investigate longitudinal trends in the utilization of diagnosis codes for “transsexualism” and “gender identity disorder.”

## 2. Methods

### Data Set

This retrospective study used publicly available data from the California Office of Statewide Health Planning and Development (OSHPD) from 2012 to 2021. The OSHPD database collects data from emergency room visits, inpatient admissions, and ambulatory surgical procedures from nearly 7,000 licensed and certified healthcare facilities across the state of California, with the purpose of promoting an equitably distributed healthcare workforce and publishing data on healthcare outcomes. The OSHPD data provides anonymized information from hospitals and EDs, including patient characteristics (residential ZIP code, sex, age, ethnicity/race), expenses and source of payment, primary diagnosis (the condition established to be the chief cause of the admission or ED encounter), and up to 24 secondary diagnoses (which could either be an active issue requiring treatment during the given specific visit or a pre-existing comorbidity). To protect patient confidentiality, the individual patient records are aggregated into data products, which are available on an annual basis. As no individual patient information was obtained, this study was deemed exempt by the Cedars-Sinai Institutional Review Board.

### Selection of relevant diagnostic codes

ED visits from 2012 to 2021 were queried for diagnosis terminology commonly associated with TG patients based on TG care guidance from several professional organizations (American Association of Coding Professions, American Association of Family Physicians, and American Psychological Association) [13,14]. This guidance was based on the International Classification of Diseases, 9^th^ (ICD-9; “transsexualism”) and 10^th^ revisions (ICD-10; “gender identity disorder”) diagnosis codes, which can have multiple clinical diagnostic terms and ICD codes [11,12]. As table 1 illustrates, in the ICD-9 list of diagnosis codes, the umbrella diagnostic term used in reference to transgender people is “transsexualism,” which reflects a sexuality-based approach to diagnostic categorization. In contrast, in the ICD-10 list of diagnostic codes, the umbrella diagnostic term used in reference to transgender people is “gender identity disorders”, which reflects a shift in diagnostic categorization to an identity-based, versus sexuality-based, approach. In both the ICD-9 and ICD-10, each of these umbrella diagnostic terms are associated with a multitude of diagnostic subtypes and codes.

### Data Analysis

Clinical diagnoses were recorded with ICD-9 nomenclature in 2012, 2013, 2014, and the first three quarters of 2015. All OSHPD institutions were required to transition to ICD-10 coding in Q4 of 2015. ICD-10 nomenclature was used from 2016 to 2021. To be able to make comparisons across classification versions, codes were grouped into equivalencies based on free text descriptions (see table 1 for equivalencies). Trends in diagnosis code usage were analyzed over the investigation period. Sub-analysis was performed on trends in usage for the primary and secondary diagnoses from 2012 to 2019 (the OSHPD database did not differentiate between primary and secondary diagnoses in 2020 and 2021). Sen’s slope and Mann-Kendall tests were used to compute the magnitude and significance of trends. Statistics were calculated using R.

## 3. Results

**Figure 1:**
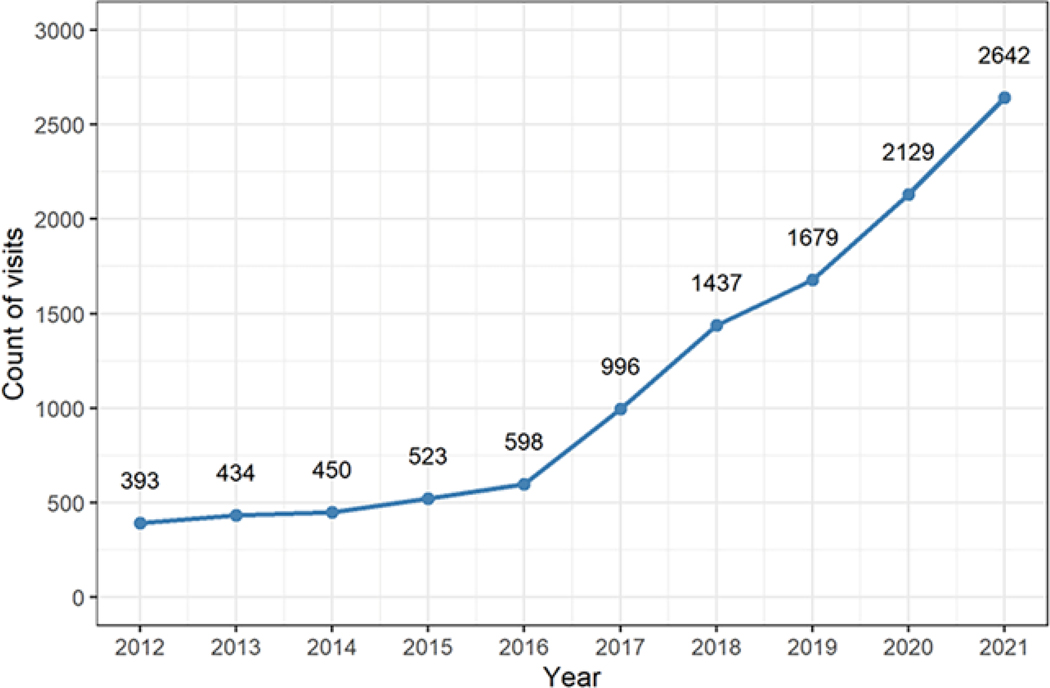
Emergency department visits per year involving a transgender-associated International Classification of Disease code

**Figure 2:**
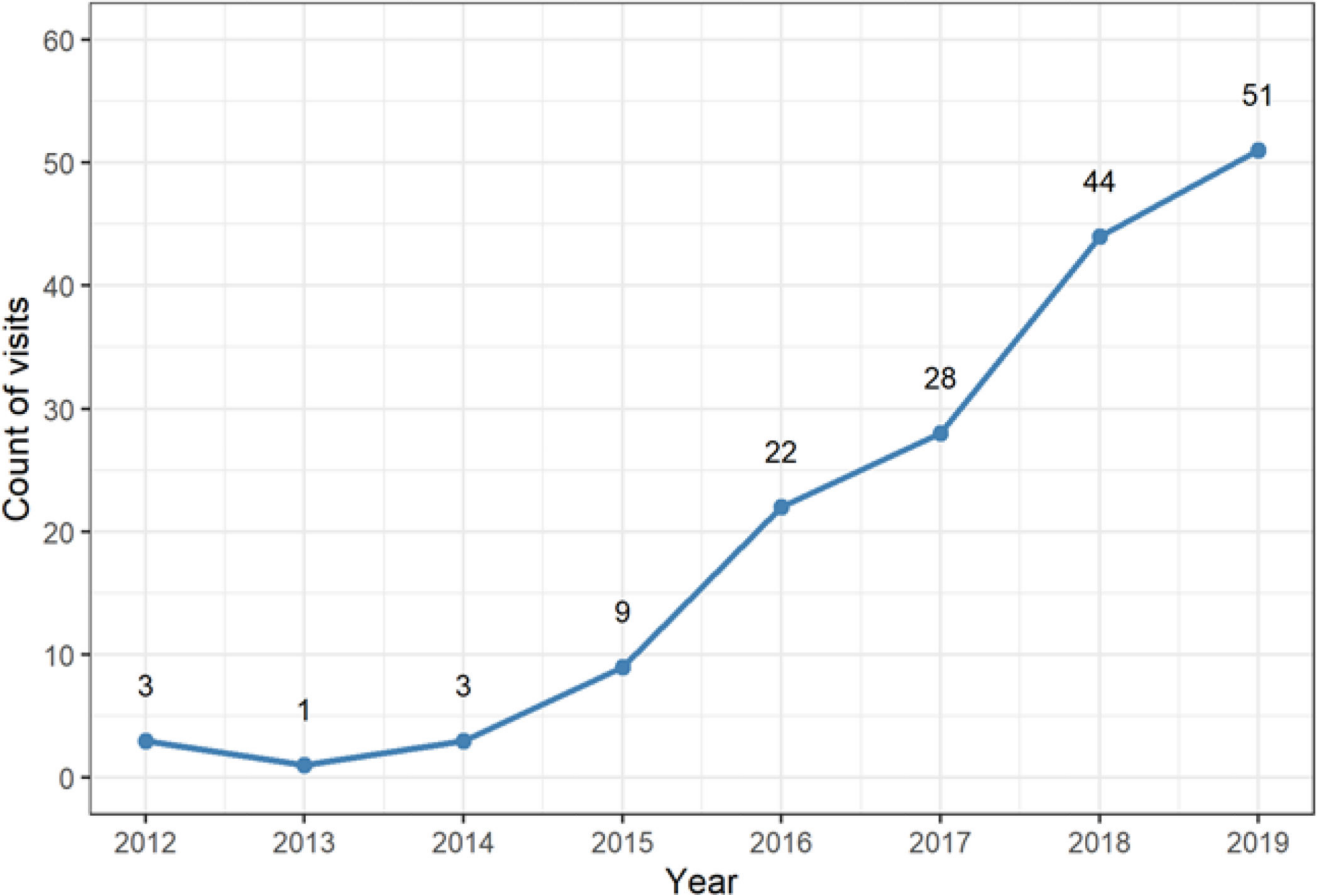
Emergency department visits per year in which a transgender-associated International Classification of Disease code was listed as the primary reason for the visit.

**Figure 3:**
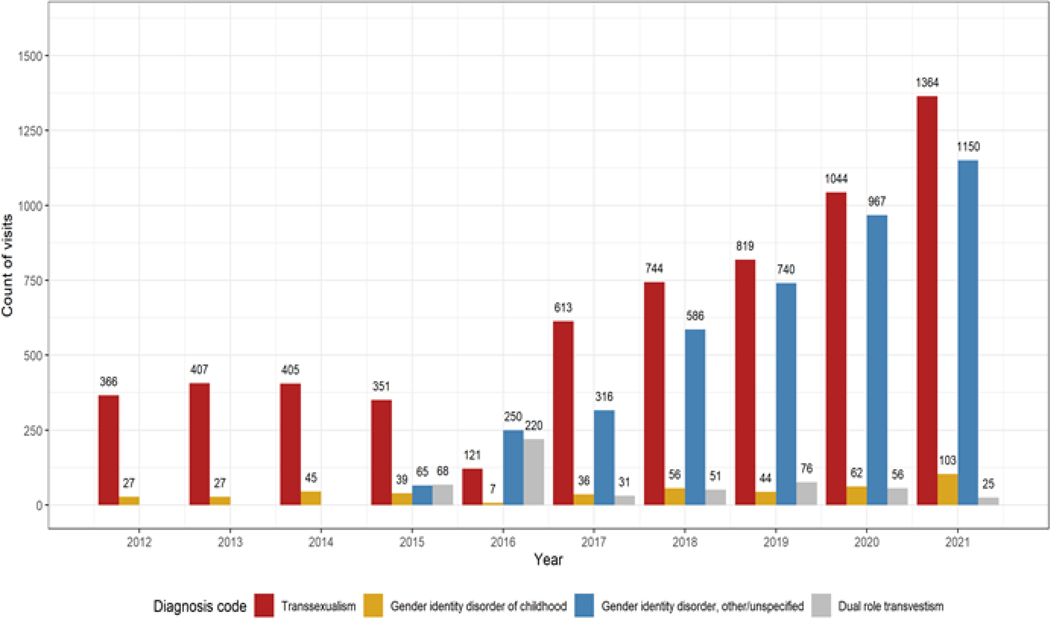
Trends in transgender-associated diagnosis code usage from 2012 to 2021. Note: The codes for “dual role transvestism,” “other gender identity disorders,” and “gender identity disorder, unspecified” were introduced in International Classification of Disease version 10, starting in Q4 2015.

In 2012, 393 out of 10.6 million ED visits (0.0037%) captured in the OSHPD database involved a diagnosis code that was associated with TG individuals. In 2021, 2642 out of 12.9 million ED visits (0.021%) involved such a diagnosis code (Figure 1). This represents a 570% increase in the number of visits with a TG-associated diagnosis code (Sen’s slope = 247 visits per year, p<0.001), despite only a 22% increase in the total number of ED visits over the ten-year investigational period. In 2012, gender transition healthcare was the primary reason for the visit in 0.76% of relevant visits (3 total), compared to 3.0% (51 total) in 2019 (Figure 2). Usage of diagnosis codes involving the term “transsexualism” decreased from 93% of relevant visits (366 total) in 2012 to 52% (1364 total) in 2021 (Figure 3). However, there was only a small relative increase in the usage of diagnosis codes involving the term “gender identity disorder” since the introduction of ICD-10, from 43% (257 total) in 2016 to 47% (1253 total) in 2021. Usage of the diagnosis code “dual role transvestism” was also highest in 2016 by a notable margin, at 37% of relevant visits (220 total).

## 4. Discussion

In this study, we used publicly available data to estimate how frequently TG patients used ED services in the state of California from 2012 to 2021. The data shows a significant 570% increase in the use of TG-associated codes over the investigational period. This increase was likely driven by both patient and provider reactions to the increased public awareness and media coverage of TG individuals. Patients may be less likely to hide that they have gender dysphoria for fear of discrimination, and providers may now be more likely to directly ask about gender identity. Despite the increase in code usage, the net documented proportion of TG patients presenting to emergency rooms in California, which was 0.021% of visits in 2021, remained much lower than would be expected based on epidemiologic prevalence studies of TG individuals in California (0.76%) [7]. Additionally, there was a significant increase in the number of visits for which a TG-associated ICD code was the primary reason for seeking medical attention. Increasing access to gender affirming surgery would likely account for a greater number of TG patients presenting for post-surgical complications. However, even in 2019, this accounted for only 3% of relevant patient encounters, suggesting that most TG patients going to the ED are seeking medical attention for issues not directly related to their transition. The utilization of TG ICD codes is rapidly rising, providing opportunities to use this method to investigate and improve healthcare outcomes for TG patients. We acknowledge the method of using relevant diagnostic codes to identify TG patients is not completely accurate, but it has been validated in other large healthcare system datasets [15–19]. Studies using ICD codes to identify transgender patients Medicare datasets report a prevalence of approximately 0.02%, which is in line with the 2021 data presented here [18,19]. However, we know this to be an underestimation of the true prevalence, when compared to studies that relied on self-reported transgender status [20,21].

Knowing the prevalence of TG patients seeking medical attention in the ED and having a patient’s gender status clearly documented in the EHR is beneficial to healthcare systems, providers, and patients. For example, prevalence data affords healthcare systems and institutions the opportunity to ensure appropriate training for staff members as well as having appropriate patient resources, screening tools, and mental health providers available for TG patients who access care via the ED. Providers not having accurate information about a patient’s birth sex or transition status can result in serious patient safety issues and potentially alter a patient’s workup and eventual diagnosis. To make correct clinical decisions, physicians need correct information regarding a patient’s natal and current physiology and anatomy [9]. EHRs that do not have easy methods of identifying a patient’s preferred gender and transition status rely on extra methods of paper documentation or constant verbal handoffs, which can stigmatize and alienate TG patients [2]. Including sexual and gender identity as a standard part of the EHR and intake questioning can improve the relationship between TG patients and providers [2]. Current medical documentation practices and regulations do not emphasize ensuring that sexual identity and gender identity fields are accurately filled, resulting in significant knowledge gaps in the disease burden and healthcare needs of sexual and gender minority patients [2]. The World Professional Association for Transgender Health (WPATH) published recommendations in 2014 for EHR developers regarding increased inclusivity for TG patients. The US government has also required EHR vendors to offer gender identity fields to be certified for meaningful use since 2013, but providers were not required to use these fields until 2016 [8,22–24].

Even with this mandate, most patients do not have gender information in their medical records. Grasso et al. assessed compliance in the first year of this mandate and found that gender identity was missing in 63% of patients, with another 9% electing not to disclose their gender information [23]. Furthermore, while “meaningful use” has specific criteria for reporting and surveillance of oncology and syndromic diagnoses, these provisions do not exist for mental health diagnoses. Thus, it is unknown if current “meaningful use” provisions will be enough to increase EHR inclusivity and visibility for TG patients. To the best of our knowledge, Centers for Medicare and Medicaid Services (CMS) do not differentiate between any of the ICD codes used in this study in terms of treatment approval or reimbursement (although it should be noted that various commercial payers may not follow CMS guidelines). In assessing observable trends, we saw clear changes in terminology used to refer to and identify patients as transgender, at least among ED providers. The decline in use of the term “transsexual,” and the increased utilization of less stigmatizing and more general terms, such as “gender dysphoria,” parallels the increased social acceptance of TG individuals and seemingly greater attention to culturally competent care. There are two likely reasons for this trend: the first is the general movement away from charged and misguided terms like “transsexual,” and the second is the reality that most ED providers are not trained to parse out the nuanced and sometimes subtle differences between “transsexualism,” “dual role transvestitism,” and “gender identity disorder.” The continued use of these more antiquated (and pathologizing) terms is likely a result of poor EHR design. In commonly used EHRs, a free text search for “gender dysphoria” results in multiple ICD-10 F64 codes for gender identity disorders, as the inclusion terms for the different sub-diagnoses overlap. The codes are listed in numerical order, which steers physicians towards choosing the first result: F64.0 or “transsexualism.” Patients who see this outdated and often inappropriate term in their medical records can feel alienated, which can exacerbate mistrust in the healthcare system and cause direct harm to patients [25].

Moreover, the F64 codes are listed under a range of “mental, behavioral, and neurodevelopmental disorders;” they were created for the DSM-V and were not designed to be used as general medical terms, nor were they designed to reflect the diverse reality of the TG community [26]. Their use has consequently led to friction between patients and well-intentioned physicians [2]. Furthermore, many emergency room providers do not have the bandwidth, or familiarity with current terminology used by TG and GNB communities, to knowledgably parse through subtle differences in these diagnoses, especially if the patient is not clearly seeking medical care related to their gender transition. All of the aforementioned suggests that we need a change in both EHR design and ICD diagnosis codes. Furthermore, it is also necessary to increase the specificity of TG-relevant ICD codes and broaden ICD codes to include people who identify as gender non-binary. These are all issues that the medical community is going to have to address moving forward.

We chose to focus our assessment on emergency room data instead of inpatient data for several reasons. First, we felt that because EDs traditionally serve a wider cross-section of social groups, including marginalized populations and those without insurance, we would have a better chance to capture a larger proportion of TG patients. Second, because there is mistrust between TG patients and the medical community, we felt that most admissions with a TG-related ICD code would most likely be directly related to the patient’s history of gender affirming surgery. Many healthcare systems have policies, enacted in an attempt to protect patient privacy, which prohibits the inclusion of a TG diagnosis code in the patient’s chart unless the reason for that specific visit or admission is directly related to their gender transition. We felt that the latter would further limit the generalizability of any conclusions gained from looking at inpatient data. Finally, TG individuals have higher incidences of high-risk behaviors and events which would result in ED visits (i.e., self-harm, substance abuse, and gender-based violence), and so, we wanted to track practice patterns in specialties that might not be aware that they participate in TG care. It is estimated that only 0.028% of a medical students first two years of instruction is spent on transgender care, but the reality is that, whether they are aware of it or not, all specialties and providers provide healthcare to TG people [26]. It falls on providers who focus on this population to work to educate colleagues about proper coding and diagnosis so that outcomes can be tracked and improved.

### Limitations

There are several important limitations of this study: First, this was a retrospective chart review that was susceptible to all the errors and inconsistencies associated with this study design. Second, because the data set captured ED visits in aggregate rather than patient specific information, there is no way of knowing if the observed increase was truly from a broader number of patients being open about their gender identity. Third, we acknowledge that there was likely a large number of TG patients who were not identified and/or accounted for with an ICD diagnosis code. Fourth, patient assigned sex at birth and gender were not clearly identifiable in the data set. For example, we could not distinguish between transgender men and transgender women, nor could we confirm whether the listed sex was the birth-sex or the gender to which the patient had transitioned. This also applied to GNB patients. We were also unable to confirm how many patients were missed because they had already fully transitioned and no longer considered themselves as having gender dysphoria. Fifth, current ICD coding does not allow differentiation between patients who identify as TG versus GNB, and current coding does not afford any indication about where any given patient is in their own process of gender transition.

### Future directions

Despite these limitations, this work represents the first attempt to assess TG healthcare needs from a non-LGBT-specific database, and it has proved to be a promising avenue of research to close the knowledge gap that exists on the healthcare needs of TG patients. Based on the success of this study, we are planning follow-up studies using patient-level data to see if having a diagnosis of gender dysphoria affects a patient’s disposition from the ED and an investigation of inpatient trends in the use of gender dysphoria diagnosis codes.

## 5. Conclusions

There was a significant increase in the TG-associated diagnosis codes in ED visits in California between 2012 and 2021. Despite this increase, such encounters were only a fraction of what would be expected based on TG prevalence estimates. There is an urgent need for more specific, descriptive, and inclusive ICD codes. There is also a need for further research into the healthcare needs of TG and GNB people (other than gender transition healthcare) so that EDs can prepare their physicians, nurses and allied health providers to provide more appropriate services to TG and GNB patients, such as disease screening and mental health resources.

## Figures and Tables

**Table 1:** ICD-9 and ICD-10 coding equivalencies used

ICD-9 (2012 to Q3 2015)	ICD-10 (Q4 2015 to 2021)
302.3 (Transvestic fetishism)	F64.0 (Transsexualism)
302.50 (Trans-sexualism, unspecified)	Inclusion term(s): Gender identity disorder in adolescence and adulthood; gender dysphoria in adolescents and adults
302.51 (Trans-sexualism, asexual)	
302.52 (Trans-sexualism, homosexual)	
302.53 (Trans-sexualism, heterosexual)	
NA	F64.1 (Dual role transvestism)
302.6 (Gender identity disorder of childhood)	F64.2 (Gender identity disorder of childhood)
	Inclusion term(s): Gender dysphoria in children; psychosexual identity disorder of childhood
NA	F64.8 (Other gender identity disorders)
	Inclusion term(s): Other specified gender dysphoria
NA	F64.9 (Gender identity disorder, unspecified)
	Inclusion term(s): Gender dysphoria unspecified; gender-role disorder NOS; identity disorder (child)
